# Fast Twist Angle Mapping of Bilayer Graphene Using Spectroscopic Ellipsometric Contrast Microscopy

**DOI:** 10.1021/acs.nanolett.3c00619

**Published:** 2023-06-08

**Authors:** Teja Potočnik, Oliver Burton, Marcel Reutzel, David Schmitt, Jan Philipp Bange, Stefan Mathias, Fabian R. Geisenhof, R. Thomas Weitz, Linyuan Xin, Hannah J. Joyce, Stephan Hofmann, Jack A. Alexander-Webber

**Affiliations:** † Department of Engineering, University of Cambridge, 9 JJ Thompson Avenue, Cambridge CB3 0FA, United Kingdom; ‡ I. Physikalisches Institut, Georg-August-Universität Göttingen, 37077 Göttingen, Germany; § Physics of Nanosystems, Department of Physics, Ludwig-Maximilians-Universität München, Geschwister-Scholl-Platz 1, Munich 80539, Germany

**Keywords:** twisted bilayer graphene, spectroscopic imaging ellipsometry, ellipsometric contrast microscopy

## Abstract

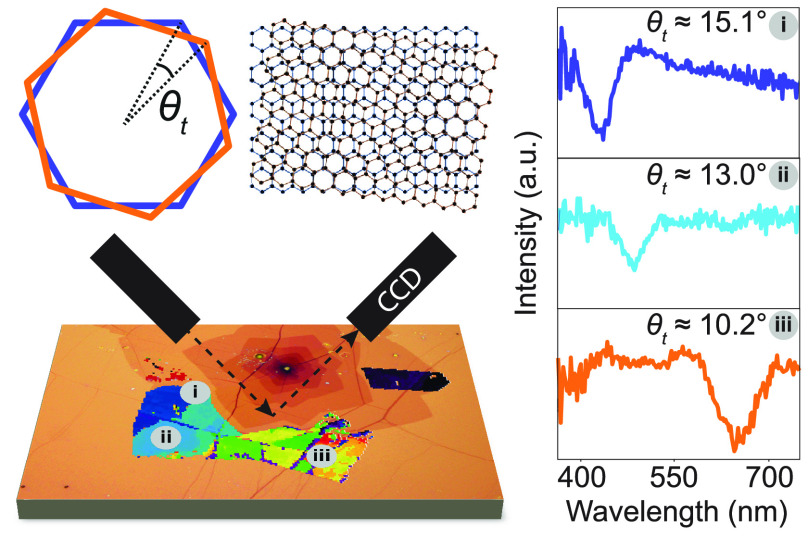

Twisted bilayer graphene provides an ideal solid-state model to explore correlated material properties and opportunities for a variety of optoelectronic applications, but reliable, fast characterization of the twist angle remains a challenge. Here we introduce spectroscopic ellipsometric contrast microscopy (SECM) as a tool for mapping twist angle disorder in optically resonant twisted bilayer graphene. We optimize the ellipsometric angles to enhance the image contrast based on measured and calculated reflection coefficients of incident light. The optical resonances associated with van Hove singularities correlate well to Raman and angle-resolved photoelectron emission spectroscopy, confirming the accuracy of SECM. The results highlight the advantages of SECM, which proves to be a fast, nondestructive method for characterization of twisted bilayer graphene over large areas, unlocking process, material, and device screening and cross-correlative measurement potential for bilayer and multilayer materials.

The band structure and thus electronic properties of twisted bilayer graphene can be tuned by the relative orientation or twist angle θ_*t*_ between the two layers. This results in novel properties such as topological transport,^1^ enhanced photocurrent,^2^ and correlated insulating phases^3,4^ at particular twist angles. Higher energy interlayer interactions can be observed such as the formation of van Hove singularities (vHs)^2,5−9^ in the electronic density of states (DOS) due to the superposition of bands from each of the two graphene layers. Optical resonances associated with transitions resonant with vHs occur with a twist angle-dependent energy separation continuously tunable from infrared (IR) to ultraviolet.^9^ This enhanced absorption has been demonstrated to be beneficial to the performance of wavelength-selective photodetectors,^2,10,11^ motivating the development of optically resonant twisted bilayer graphene for optoelectronic devices.

Twisted bilayer graphene can be created directly during growth,^12,13^ by stacking graphene monolayers^14−16^ or by folding them using atomic force microscope (AFM) tips.^17^ Any contamination between layers during processing reduces the interlayer coupling which may destroy angle-dependent phenomena.^5^ Atomically clean interlayer interfaces can be found in as-grown chemical vapor deposition (CVD)^18,19^ bilayer graphene.^20^ Significant effort has been made to tailor CVD growth parameters to selectively obtain bilayer graphene,^21^ which is typically hindered by screening of carbon precursors from the growth catalyst by the primary graphene monolayer. A further challenge, specific to direct growth of twisted bilayer graphene,^12^ is avoiding formation of the energetically favorable AB stacked and 30° rotated configurations which often make up the majority of CVD grown bilayer and multilayer graphene.^20,22^

Identifying bilayer graphene with a particular twist angle is challenging, particularly substrate-agnostic, fast, large-area mapping. Angle-dependent characterization of twisted bilayer graphene has been demonstrated through optical absorption^6,23^ and reflection,^24−26^ photoemission,^2,12,27−29^ photoluminescence,^7,30^ and Raman^5,13,31,32^ spectroscopies, which are often correlated with higher resolution electron microscopy^20,25^ or scanning probe microscopy.^33−36^ Many of these techniques rely on specific substrate properties, such as transparent^6^ or contrast-enhancing,^24,37^ which may be incompatible with characterization during particular stages of manufacturing.^38^ Spectroscopic imaging ellipsometry has emerged as a tool to determine the optical constants of graphene^39^ and other two-dimensional materials^40^ and provide thickness information with single-atomic layer precision^41,42^ with a lateral resolution down to ∼1 μm on a wide range of substrates, including as-grown directly on metal catalyst foils.^43^ Spectroscopic ellipsometry measures the change in polarized light upon reflection at a sample to determine the wavelength-, λ, dependent complex dielectric function (or refractive index *n*(λ) and extinction parameter *k*(λ) values) which provides insight into fundamental light–matter interactions crucial for understanding a range of optical phenomena.^44^ To extract the full complex dielectric function or layer thicknesses of a given sample the ratio of perpendicular *p* and orthogonal *s* components of the reflected light represented by ρ = *R*_*p*_/*R*_*s*_ = tan(*ψ)e*^*i*Δ^, where ψ is the amplitude ratio and Δ is the phase difference, is measured as a function of λ, angle of incidence (AOI), and polarizer (P), analyzer (A), and compensator (C) angles, and fitted to an optical model.^44^

Alternatively, ellipsometer settings (AOI, P, A, C) can be optimized for material and thickness contrast and fixed during imaging. This technique, often termed ellipsometric contrast microscopy (ECM), has been used across a variety of fields^43,45,46^ and only images the intensity of reflected light, significantly improving characterization throughput.^43^ Building on this technique, here we perform spectral ECM (SECM) of the chemical vapor deposition (CVD) grown monolayer, bilayer, and multilayer graphene transferred onto Si/SiO_2_. We demonstrate that, in addition to layer-number sensitivity and high material contrast, SECM provides wavelength-dependent contrast of optically resonant bilayer and multilayer regions allowing us to extract a map of the twist angle variation. The range of twist angles detectable is set by the spectral range of the image sensor. We validate the technique by correlating SECM data with Raman mapping and angle-resolved photoelectron emission spectroscopy (ARPES).

Figure 1a shows an optical micrograph of two merged bilayer and multilayer islands. Several bilayer grains within a 1 cm^2^ transferred graphene film showed colored regions in optical microscopy which typically appear as approximately radial sections in one or more “petals” within the bilayer graphene “flowers”. The region shown in Figure 1a was selected for further study as it showed blue, green, yellow, and red hues in close proximity to one another. Such colored regions are associated with enhanced absorption consistent with vHs-resonant transitions,^9^ as schematically shown in Figure 1b. SECM reveals that a rich variety of twist angles can be observed in this region as described below.

**Figure 1:**
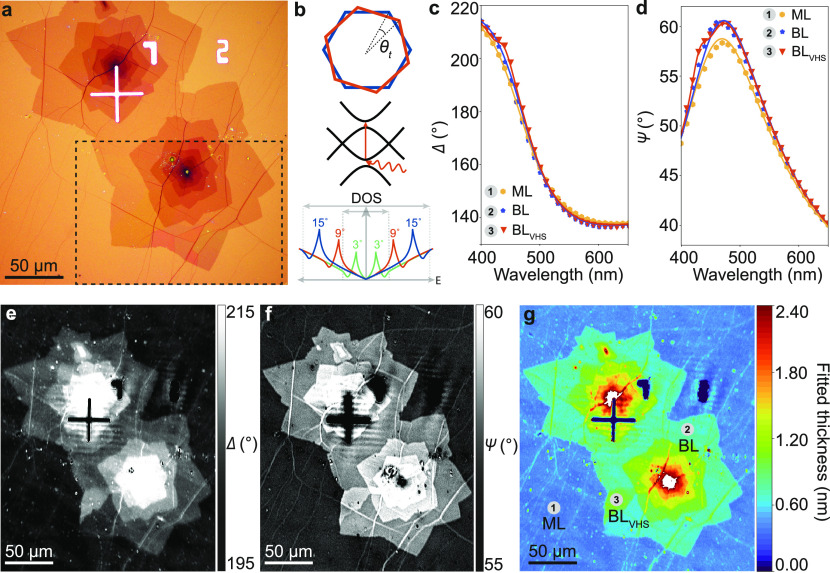
(a) Optical microscopy image of a graphene flake on the Si/SiO_2_ substrate. Dashed square represents the region studied in SECM and Raman spectroscopy. (b) Schematic showing the Brillouin zone and twist angle θ_t_, band structure for twisted bilayer graphene, and density of states of twisted bilayer graphene at different twist angles with the horizontal lines corresponding to the highest and lowest energy transitions for the wavelength range studied. Fitting of Δ (c) and ψ (d) of the monolayer (ML), bilayer (BL) and resonant bilayer (BL_VHS_) to the Si/SiO_2_/graphene (+ Gaussian) layer stack model. Imaging RCE mapping of Δ (e) and ψ (f) at λ = 440 nm. (g) Graphene thickness map of the flake at λ = 440 nm as fitted using a Si/SiO_2_/graphene model, without accounting for resonances.

To extract layer thickness for the graphene flake we perform imaging ellipsometry using a rotating compensator ellipsometry (RCE) method to measure ψ and Δ as a function of λ. We used the imaging RCE mode from 400 to 650 nm over 10 nm intervals, where a spatial map of Δ and ψ is measured for each wavelength interval, yielding 25 maps for both Δ and ψ. P and A were both fixed at 45°, with AOI at 50°. Examples of Δ and ψ maps at λ = 440 nm are shown in Figure 1e,f, respectively. We then performed map analysis to extract data averaged over the predefined region of interest for maps at all wavelengths within the range. We chose three different regions based on the optical microscope image in Figure 1a: monolayer (ML), bilayer (BL), and resonant bilayer (BL_VHS_), as labeled in Figure 1g. Figure 1c,d show the measured Δ and ψ, respectively, as a function of wavelength for the three regions of interest. We fitted the wavelength dependence of Δ and ψ for ML and BL with the Si/SiO_2_/graphene model described in the Methods section (see Supporting Information). To account for the presence of vHs, a Gaussian resonance term was added to the graphene model when fitting to the data extracted from BL_VHS_. The thickness of graphene *t*_*Gr*_ was found to be 0.57 ± 0.1 nm for monolayer and 0.75 ± 0.1 nm for bilayer, as detailed in Table S1 (see Supporting Information). Despite these values deviating from the expected monolayer thickness of 0.335 nm,^47,48^ these values fall within the reported thickness deviations for copper foil-transferred graphene as measured with AFM^49−51^ and ellipsometry,^41,52^ likely due to polymer contamination or chemical interactions between graphene and the substrate.^49,53^ It can be seen in both Figure 1c,d that the resonant bilayer region curve deviates from the monolayer and bilayer regions. For the resonant bilayer we observe an additional peak in both Δ and ψ curves centered at approximately 440 nm. Spatial maps of Δ and ψ (Figure 1e,f) show that the increased values at λ = 440 nm are consistent across a resonant bilayer region. Figure 1g shows a spatial map of the fitted graphene thickness determined from Δ and ψ values for a single wavelength (λ = 440 nm) where each pixel of the map was fitted with a Si/SiO_2_/graphene layer stack, i.e., without an additional Gaussian term, where the only free parameter was *t*_*Gr*_. We see that under resonant illumination the extracted values of *t*_*Gr*_ for the region BL_VHS_, fitted using the simple graphene model, deviates from the adjacent bilayer regions, showing a higher apparent thickness.

To find the maximum contrast between monolayer graphene and resonant bilayer graphene on Si/SiO_2_ for ECM, we evaluate the choice of ellipsometer parameters, as shown in Figure 2. It has been shown that ellipsometric angles for optimized contrast rely heavily on the number of graphene layers, as well as the choice of substrate and range of wavelengths.^43^Figure 2a shows examples of ellipsometric contrast images as a function of AOI centered on another optically resonant bilayer region under λ = 480 nm, for P = A = C = 0°. We observe the strongest contrast for the resonant bilayer at AOI = 40°. We calculate the measured image contrast between the monolayer and bilayer regions using the Weber relation where contrast = , and *I*(*ML*) and *I*(*BL*) are the average pixel intensity values of the monolayer background and the BL or BL_VHS_, respectively. We theoretically model this by calculating the reflection coefficient *R*_*p*_ (Figure 2b) as a function of AOI using the model parameters determined from the fitting results described above (Table S1), where *R*_*p*_ = 1 corresponds to 100% reflected intensity. For the resonant bilayer region, the measured image contrast follows the theoretically expected contrast . For the nonresonant bilayer graphene region, the contrast is underestimated by the model, as the model does not account for nonidealities such as contamination (which increases the thickness of the layers). To further investigate the choice of optimum contrast for observing resonant bilayer graphene, we measure contrast at AOI = 40° and as a function of P and A for the resonant bilayer (λ = 480 nm) at two different C angles, C = 0° and C = 45°. The measured contrast for the resonant bilayer is shown in Figure 2c. It can be seen that the maximum contrast can be achieved when the polarization of light is parallel to the surface plane, with P = 90°, A = 90°, and C = 0°. We use these optimized parameters for the wavelength-dependent mapping described below.

**Figure 2:**
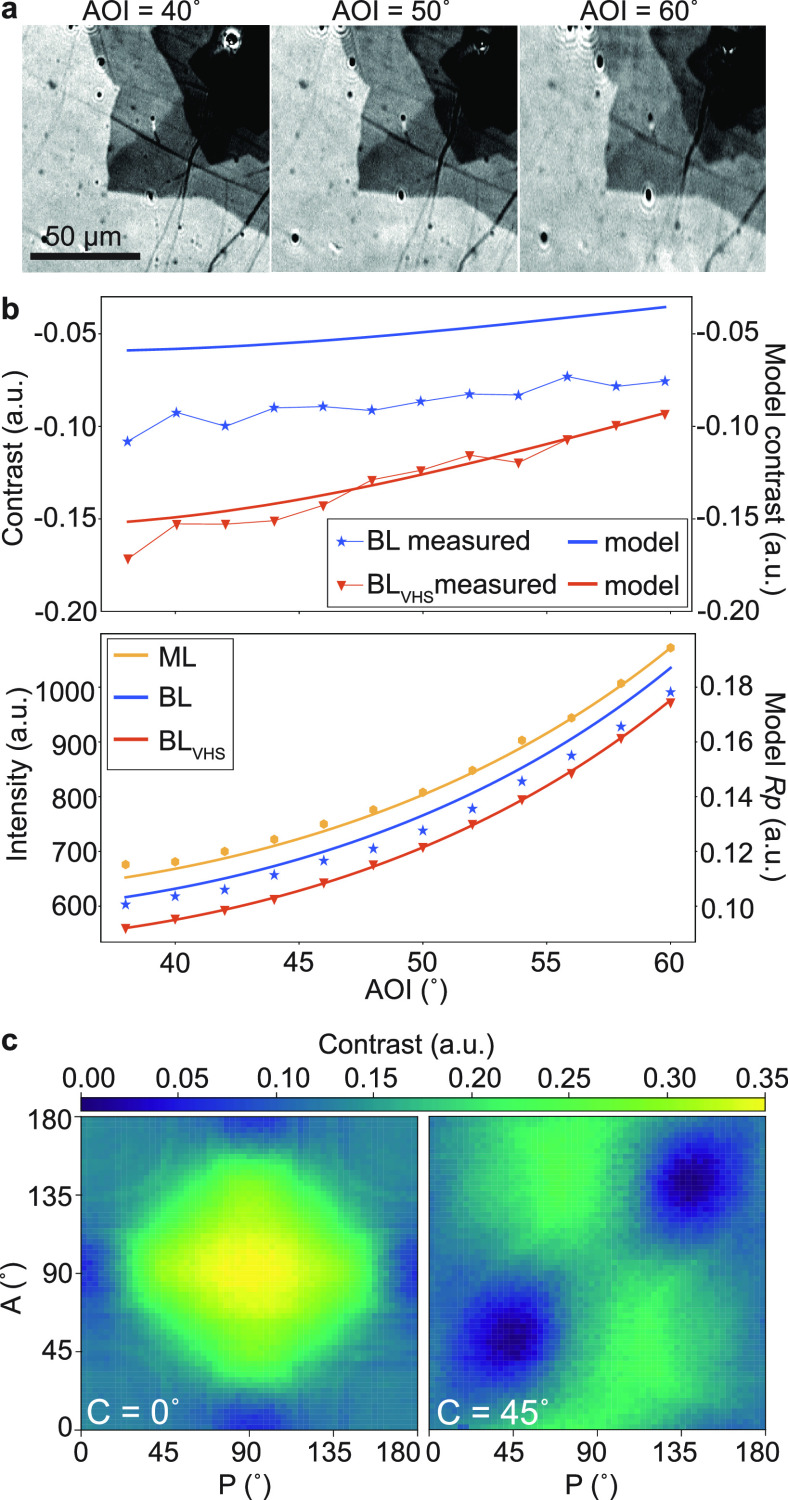
Optimising ellipsometer parameters for bilayer graphene on Si/SiO_2_. (a) ECM images of graphene flake at different angles of incidence (AOI). (b) *R*_*p*_ coefficient as a function of AOI with calculated Weber contrast. (c) Normalized intensity as measured for P and A rotation on Si/SiO_2_/graphene at C = 0° (left) and C = 45° (right).

Figure 3a shows the *R*_*p*_ and *R*_*s*_ reflection coefficients as a function of wavelength for monolayer (ML), bilayer (BL), and resonant bilayer (BL_VHS_) regions as labeled in Figure 1g, with the corresponding Weber contrast calculated for bilayer and resonant bilayer regions with a background of monolayer graphene. The *R*_*p*_ and *R*_*s*_ coefficients were calculated using the layer-stack model parameters described above with AOI = 40°, with an additional Gaussian resonance centered at the corresponding wavelength of 440 nm applied to resonant bilayer region data. There is deviation noticeable in the reflection coefficient for the resonant bilayer region as compared to monolayer and bilayer regions. This results in a predicted enhancement of the contrast on resonance for BL_VHS_. We perform SECM on the bilayer graphene regions shown in Figure 1a. Under fixed ellipsometer angles we image the reflected intensity as a function of wavelength varied from 350 to 750 nm. To focus specifically on bilayer graphene, we implement a mask based on average pixel intensity to remove data points corresponding to areas of monolayer, or multilayer (≥3 layers), graphene, as indicated in Figure S1 (see Supporting Information). The spatial distribution of the resonant wavelength within the bilayer regions is shown in Figure 3b, with annotations for corresponding spectra at these regions shown in Figure 3c. We use (54) to determine the twist angle from the resonant wavelength (Figure 4a, inset), with Fermi velocity *v*_*F*_ = 1 × 10^6^ m s^–1^, ℏ the reduced Planck’s constant, and *a* = 2.46 Å the graphene lattice constant.

**Figure 3:**
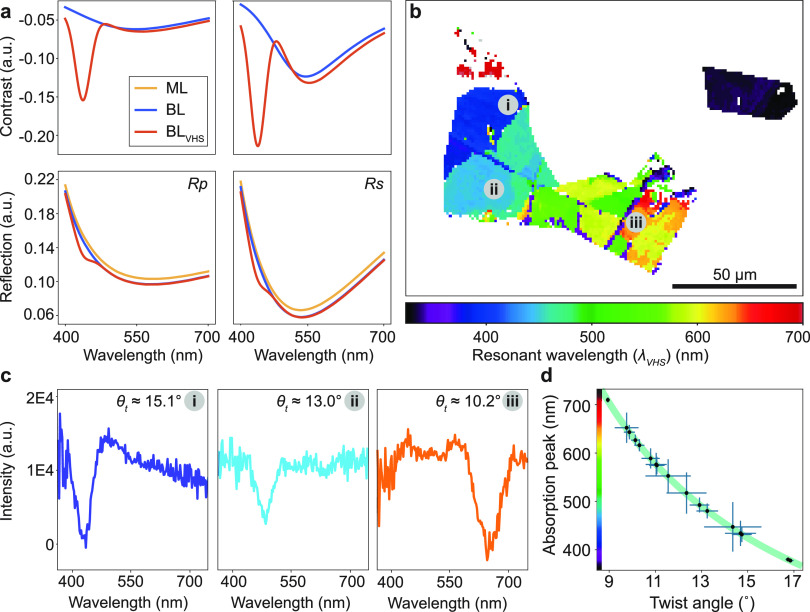
(a) *R_p_* and *R_s_* reflection coefficients as a function of wavelength for monolayer (ML), bilayer (BL), and resonant bilayer (BL_VHS_) regions as labeled in Figure 1g with calculated Weber contrast above each plot. (b) Resonant wavelength map of graphene extracted by fitting a Gaussian peak to the intensity as a function of wavelength at each pixel measured in SECM. (c) Reflected intensity from SECM as a function of wavelength showing absorption resonances for the regions marked in (b) (i–iii). (d) Absorption peak as a function of twist angle.

**Figure 4:**
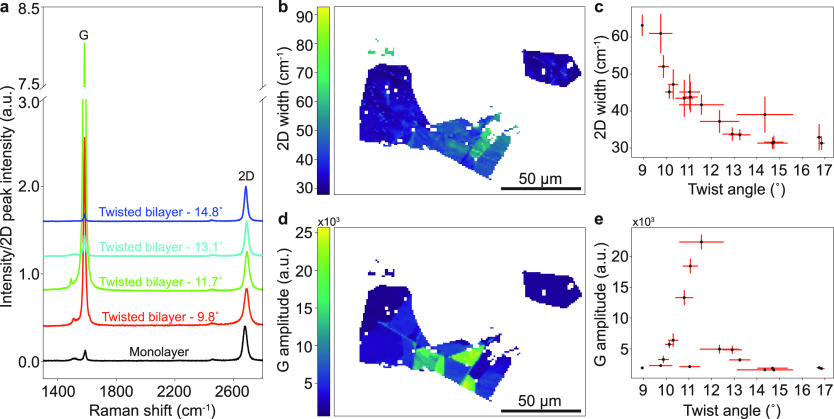
(a) Raman spectra of graphene at different twist angles, normalized with respect to 2D peak intensity. (b) Raman 2D-peak width map of graphene. (c) Raman 2D-peak width of graphene as a function of bilayer twist angle. (d) Raman G peak amplitude map of graphene. (e) Raman G-peak amplitude as a function of bilayer twist angle. The error bars on the plots represent the standard deviation.

To validate our methodology, we correlate the twist angles determined from SECM with Raman spectra. The individual Raman spectra for different twist angle bilayer regions, compared to a reference monolayer region, are shown in Figure 4a. The Raman spectra show G and 2D peaks at ∼1580 cm^–1^ and ∼2700 cm^–1^, respectively, and their shapes and positions, as well as intensity ratios that vary with the number of graphene layers and twist angle.^55^ The spectra correlate well with spectra from Figure 3c, which indicates that SECM data can aid the interpretation of Raman spectra. We also show a map of the graphene flake showing 2D width and G amplitude intensity (Figure 4b,d, respectively). Using image registration with the SECM data we apply the same mask to the Raman data to plot the resonant bilayer regions identified above. The widths of the 2D peaks decrease with larger bilayer twist angles, from 64 cm^–1^ for small twist angles to 30 cm^–1^ for larger twist angles, as also shown in Figure 4c. Similarly, we observe a variation in G peak intensity as a function of graphene bilayer twist angle, with a significant enhancement in G peak intensity^5^ for bilayer graphene with twist angle close to 12° (Figure 4e). This corresponds to resonant absorption at 540 nm ±20 nm, which correlates well with resonant absorption from the Raman excitation wavelength (λ = 532 nm). The regions showing G peak resonance, shown in Figure 4d, were found to spatially correlate with the regions that show resonant contrast enhancement in ECM under similar wavelengths. The *x* (*y*) error bars in Figure 4c,e correspond to the standard deviation of the twist angle (Raman feature) measured within a particular spatially localized region.

The twist angle variation in bilayer graphene is often attributed to formation during graphene growth^12^ or at wrinkles or folds within bilayer graphene films, which are one-dimensional defects that form as a result of mismatch in thermal expansion coefficients of graphene and the substrate.^56−58^ To investigate the origins of twist angle domains, we perform AFM and IR scanning near-field microscopy (SNOM) on a region showing changes in stacking between resonant bilayer regions, and which showed lines of dark contrast in the optical microscopy indicating the presence of folded graphene (Figure 1a).

Figure 5a shows the AFM map of the studied graphene flake region, with the bright lines indicating the folded bilayer graphene. We examine the AFM height profile across one of the folds as indicated in Figure 5a and find the thickness at the center of the wrinkle of 1.2 nm, indicating 4 additional layers of graphene at that region (Figure 5b). The folds in the graphene flake can also be seen in the IR SNOM^59^ as shown in the map of the second harmonic amplitude (s_2_) (Figure 5c), indicating enhanced scattering at these folds. The features in the SNOM amplitude at these twist angles (∼10°–12°) are colocated with topographic features observed in the AFM, such as folds and wrinkles.

**Figure 5:**
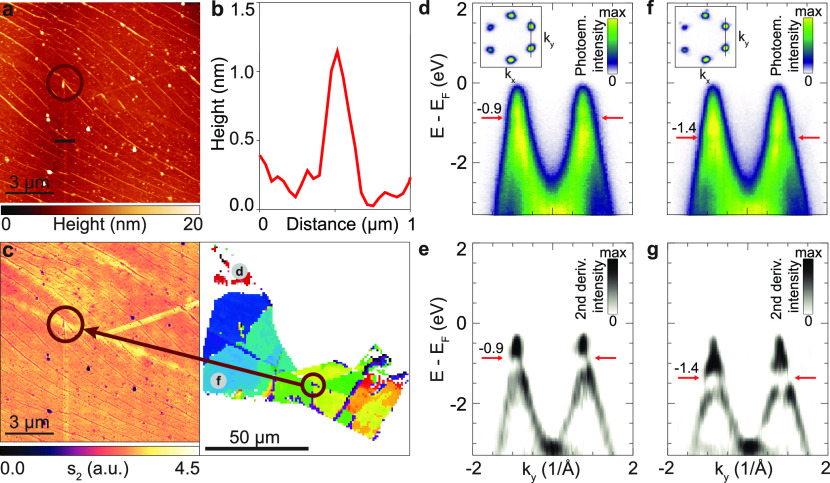
(a) AFM map of the graphene flake on Si/SiO_2_ substrate indicating the region marked in (c). (b) AFM profile of graphene across the wrinkle in the AFM map in (a), indicating four layers of graphene. (c) Map of the second harmonic (s_2_) IR SNOM signal and the corresponding resonant energy map of the graphene flake showing different twist angle domains of graphene with annotations of the studied areas. (d, f) ARPES data obtained in the regions-of-interest indicated by circles in (c). In the inset, momentum–momentum cuts are shown for energies close to the Fermi level. The energy-momentum cut is taken along the black line indicated in the insets. (e, g) The vHs in the valence band can be identified when the second derivative along the energy axis is applied to the data (energy position highlighted by red arrows). Spectrum along the k_*y*_-direction, as shown in the inset momentum map, showing the band structure of twisted bilayer graphene regions with *E*_*c*_ = 1.77 eV (d) and *E*_*c*_ = 2.70 eV (g) as determined by SECM. The corresponding integrated intensity spectra ((e) and (g), respectively) each show a resonant feature at ∼*E*_*c*_/2 (arrow).

We performed ARPES experiments to quantify the electronic band structure of twisted bilayer graphene. This experiment is especially helpful to identify the energy position of the vHs in the valence band and thus to corroborate the resonant energies obtained from ellipsometry for various twist angles. To determine the energy–momentum dispersive band structure around the vHs of different bilayer graphene regions, we use time-of-flight momentum microscopy.^60–6150^ This specialized ARPES setup is capable of probing the band structure with a spatial resolution of down to 10 μm, which allows spatial mapping of the twist angle.^62^Figure 5d,f shows energy–momentum and momentum–momentum (inset) cuts through the ARPES data taken at the two regions of interest indicated by circles in Figure 5c. In both areas, we find that the bilayer graphene flake is minimally doped, i.e., the Fermi-level is positioned within 100 meV of the Dirac point. Moreover, we only detect photoelectrons originating from the top layer (i.e., six Dirac points in the momentum–momentum cuts).

To evaluate the energy–momentum dispersive band structure and, in particular, the energy position of the vHs of the valence band in more detail, we apply the second derivative along the energy axis to the energy-momentum maps (Figure 5e,g). This data handling directly identifies abrupt changes in the photoemission intensity that we attribute to the energetic position of the vHs (highlighted by arrows). For the two regions in Figure 5e,g, we find that the vHs are located at *E* – *E*_*F*_ = −0.9 ± 0.1 and −1.4 ± 0.1 eV, respectively. Having identified the energetic position of the vHs and measured that the bilayer graphene samples are minimally doped, we can determine the energy difference between the vHs of the valence and the conduction band to 1.8 ± 0.2 eV and 2.8 ± 0.2 eV, respectively. These energies match the optical experiments shown in Figure 3b and thus fully support the application of SECM as a technique for the fast identification of the twist angle in bilayer graphene.

In this work we perform SECM and correlated characterization to study the angle dependence of optically resonant twisted bilayer graphene. We compare the SECM results to full wavelength-dependent Δ and ψ maps obtained through imaging RCE to confirm the thickness of the bilayer regions. The ellipsometer wavelength range allows us to find twist angles between 9° and 17° and provides a typical twist angle accuracy of <1°. The range of twist angles could be expanded by using a spectroscopic imaging ellipsometer with an extended wavelength range such as commercial systems capable of measuring wavelengths of 190 to 2700 nm which would correspond to θ_*t*_ ∼ 30° to ∼2°, respectively. The SECM findings agree well with ARPES and Raman characterization. While Raman measurements typically require a contrast-enhancing substrate, like the 90 nm SiO_2_-on-Si substrates as used in this work, ellipsometry is substrate agnostic, which means it is applicable to a wider range of materials, and could also be used to screen different stages of the manufacturing process. For example, high contrast is observed between monolayer and bilayer graphene—imaged as-grown on Cu or after transfer onto Si—regardless of substrate (Figure S3). This capability enables wafer-scale mapping and fast identification of bilayers even on substrates without contrast enhancement. The origin of some regions of twist-angle disorder is attributed to graphene folds as confirmed by AFM and SNOM, whereas other regions show twist angle variations away from any obvious folds. This indicates that a combination of processes during growth and postgrowth (e.g., during cooling or transfer) are responsible for the twist angle disorder. The presence of optical resonances is confirmed with ARPES by analyzing the band structure at regions with different resonant wavelengths. This technique could be applicable to other material systems,^42^ including transition metal dichalcogneides^63^ and twisted heterostuctures.^64^ With the advent of spectroscopic microellipsometry extensions for conventional optical microscopes, SECM has become even more affordable and accessible.^65^ This work confirms that SECM is a powerful, fast, and nondestructive tool for material characterization, which unlocks the potential of material properties for a variety of research applications.

## Data Availability

Datasets related to this publication are available from the Cambridge University data repository at https://doi.org/10.17863/CAM.97020.

## Supporting Information Available

Methods, fitted parameters, intensity thresholding, comparison of techniques, and bilayer graphene on Cu and Si (PDF)
