# Supplementary material for: Fast Twist Angle Mapping of Bilayer Graphene Using Spectroscopic Ellipsometric Contrast Microscopy
Source: Nano Lett. 2023 Jun 8;23(12):5506–13. doi: 10.1021/acs.nanolett.3c00619 (PMC10311600; doi:10.1021/acs.nanolett.3c00619)
Supplement: Supplementary file 1 — nl3c00619_si_001.pdf [file nl3c00619_si_001.pdf]

Supporting Information: **Fast twist angle mapping of bilayer graphene using spectroscopic ellipsometric contrast microscopy**

Teja Potočnik[1]*, Oliver Burton[1]*, Marcel Reutzel[2], David Schmitt[2], Jan Philipp Bange[2], Stefan Mathias[2], Fabian R. Geisenhof[3], R. Thomas Weitz[2,3], Linyuan Xin[1], Hannah J. Joyce[1], Stephan Hofmann[1#], Jack A. Alexander-Webber[1#]

[1] Department of Engineering, University of Cambridge, 9 JJ Thompson Avenue, Cambridge CB3 0FA, United Kingdom

[2] I. Physikalisches Institut, Georg-August-Universität Göttingen, 37077 Göttingen, Germany

[3] Physics of Nanosystems, Department of Physics, Ludwig-Maximilians-Universität München, Geschwister-Scholl-Platz 1, Munich 80539, Germany

*Equal contributions

[#]Email: sh315@cam.ac.uk, jaa59@cam.ac.uk

Methods

Graphene growth: Graphene was grown by chemical vapour deposition in a commercial Aixtron 4" BM Pro reactor on 25 µm Cu foil (98% Alfa Aesar). The procedure is outlined in Burton *et. al*,[1] where the pre-growth hydrogen exposure phase was omitted to maximize the coverage of bilayer graphene on the Cu foil. The graphene was wet transferred onto a Si wafer with 90 nm of thermally grown SiO_2. The wet transfer involved coating the graphene/Cu in a 495k A4 PMMA resist, using a O_2 reactive ion etch to remove graphene on the back side of the Cu foil, etching the Cu foil in 0.1M ammonium persulphate solution, subsequent rinsing in deionised water and finally deposition on the target Si/SiO_2 substrate.

Ellipsometry: All ellipsometry measurements were taken using a commercial Accurion EP4 ellipsometer in RCE configuration, using a 20× objective. All measurements were carried out at room temperature under ambient conditions unless otherwise specified. The model fitting was performed using the graphene/SiO2/silicon model as constructed from Accurion EP4 software dielectric function data for selected materials. We initially measured the bare substrate to determine the thickness of the SiO_2 (t_{SiO2} = 90.5 nm) to use as a fixed parameter when fitting the graphene layer. The Gaussian resonance term is defined by $Im\,(\varepsilon) = A\left(\exp\left(-\frac{(E-E_C)^2}{\sigma^2}\right) - \exp\left(-\frac{(E+E_C)^2}{\sigma^2}\right)\right)$, where $\sigma = \frac{B}{2\sqrt{\ln 2}}$, with A being the amplitude, B the bandwidth, E is the photon energy E_C the photon energy on resonance. Spectral ellipsometric contrast mapping (SECM) was conducted with an Accurion EP4 Ellipsometer with a variable wavelength source at 20× magnification. Polariser and analyser angles were determined by maximising the magnitude of the Weber contrast of a bilayer region relative to monolayer (see **Figure 2**). Monochrome images were then acquired for a range of wavelengths of incident light. This set of images were converted into a map of spectra, such that a spectrum was obtained for each spatial pixel. The median average bilayer region background was then subtracted along with a general Gaussian background removal to produce the spectra analysed in this work. For bilayer graphene analysis the data was masked to only include bilayer regions clear of alignment markers and the noise that they introduce. These bilayer spectra then had all local maxima identified, filtered by peak height and width to only include spectra with appreciable absorption peaks. The absorption peak was then defined by the most prominent peak within this subset. The twist angle was

extrapolated from the wavelength of this absorption peak as described above. Measurement time depends on a range of criteria, such as spectral resolution, spectral range, accumulation time and spatial resolution needed. A typical full image acquisition at a single wavelength, including focal line scanning, took approximately 500 ms. For 200 images at different wavelengths the total measurement time was less than 5 minutes, creating a hypercube with $>10^8$ total datapoints. The comparison of SECM with Raman and spectroscopic imaging ellipsometry (RCE mode) is given in **Figure S2**.

The SECM map and Raman map were combined through image registration. Key points on each map (such as edges, corners, and central points) were identified and matched. These key points were then used to find a perspective transform using the random sample consensus (RANSAC) method, which provided a map function enabling the association of the Raman spectra with the SECM derived spectra. After data association K-means clustering was used to sperate the data into distinct groups based on fitted characteristics from the Raman spectra (G and 2D peak heights, positions, and widths) and SECM absorption peaks. These groups were identified as spatially localised by including the normalised map coordinates in the k-means fitting process. The identified segments were then analysed to give the mean and standard deviation of various parameters, as shown in the main text. In order to provide high quality data, groups with a coefficient of variation greater than 0.2, that did not have identifiable Raman 2D and G peaks, or spectral absorption peaks, were discarded to reduce noise in the presented data.

Raman: Raman spectroscopy was performed using a 532 nm laser excitation with an exposure time of 0.1s and laser power in the order of 1mW through a 20× objective measured with a grating of 1800 l/mm and in a Renishaw inVia Raman system.

SNOM: SNOM imaging was performed on a scattering-type scanning near-field microscope (s-SNOM, neaspec GmbH) in tapping mode using infrared CO_2 laser excitation (wavelength of 10.5 µm), as described in Ref. [2].

ARPES: Before the ARPES experiments, the sample was transferred into the ultra-high-vacuum chamber and annealed for 1-2 hours at a temperature of 690 K in order to remove surface contamination. The ARPES experiments were performed with a time-of-flight momentum microscope[3] operated at a high-harmonic generation beamline delivering 20 fs 26.5 eV pulses at a repetition rate of 500 kHz.[4,5] The experiment collected multi-dimensional data sets, where the photoemission intensity was resolved as a function of photoelectron energy and two in-plane momenta (k_x and k_y). A microscope-type photoelectron analyzer collected ARPES data on sample areas with a spatial resolution of 10 µm.[6] For the data shown in **Figure 5**d and e, the energy and momentum resolution is limited by charging effects due to the substrate's oxide layer (90 nm SiO_2),[7] which may also hinder the direct observation of the Dirac cone of the lower layer.

Fitted Parameters

The fitted parameters were determined from wavelength dependent values of Δ and ψ shown in **Figure 1**c and d. The Si/SiO_2/graphene was used to determine the thickness of graphene, with an additional Gaussian term added for the resonant bilayer graphene region. The parameters are shown in **Table S1**. The Gaussian resonance term is defined by $Im\ (\varepsilon) = A\left(\exp\left(-\frac{(E-E_C)^2}{\sigma^2}\right) - \exp\left(-\frac{(E+E_C)^2}{\sigma^2}\right)\right)$, where $\sigma = \frac{B}{2\sqrt{\ln 2}}$, with *A* being the amplitude, *B* the bandwidth, *E* is the photon energy E_C the photon energy on resonance.

Table S1: *Fitted parameters for the regions of interest of graphene on SiO_2.*

	**Monolayer region**	**Bilayer region**	**Resonant bilayer region**
**Thickness (nm)**	0.57	0.75	0.75
**A**	0	0	2.625
**Ec**	N/A	N/A	2.577
**B**	N/A	N/A	0.1985

Intensity Thresholding

Figure S1 shows example threshold limits for removing datapoints using a mask based on average pixel intensity that correspond to areas of monolayer, multilayer (>3 layer) graphene.

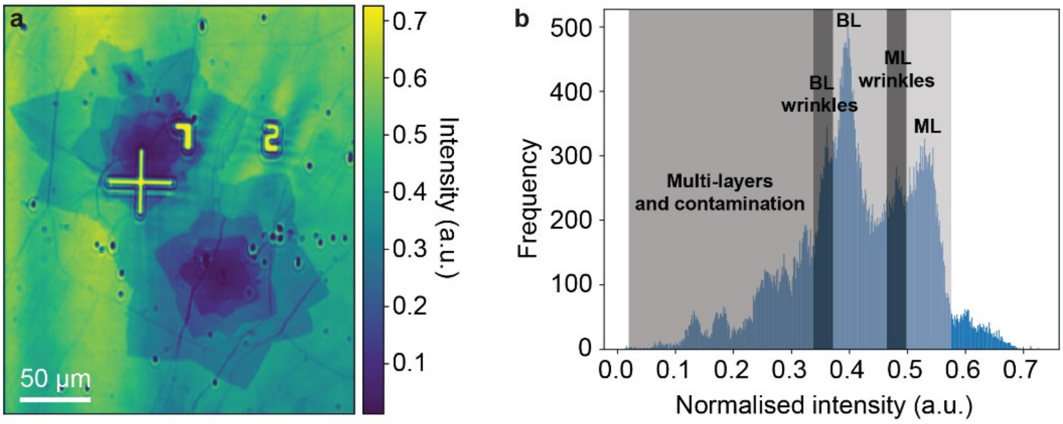

Figure S1: ***a*** *Reflected intensity map of graphene on SiO_2/Si.* ***b*** *Histogram showing normalised intensity for different graphene layers and wrinkles.*

Comparison of Techniques

In point-by-point techniques such as Raman, the acquisition of data takes a long time, but the fitting is relatively quick and simple. In imaging spectroscopic ellipsometry, acquisition depends heavily on the settings, but can be in the order of hours for multiple wavelengths. The limiting factor is the traditional modelling approach, which when applied to our dataset of 0.5 megapixels can reach 72h of fitting for a single wavelength and a single free parameter, e.g. t_{Gr} as shown in **Figure 1**g of the main manuscript. **Figure S2** shows a comparison of SECM with Raman and conventional ellipsometry, highlighting the advantages of faster acquisition and analysis achievable with SECM.

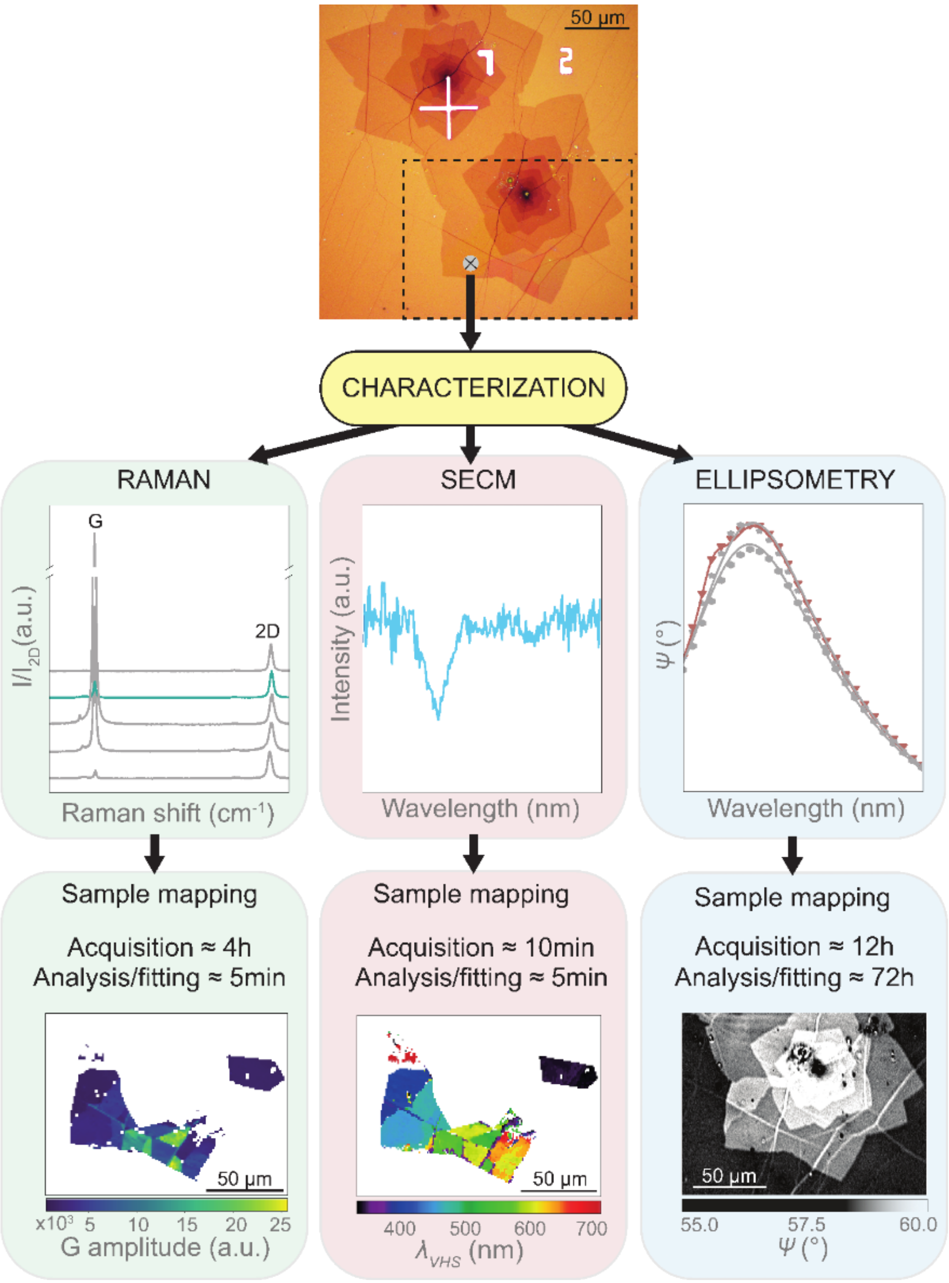

***Figure S2**: Diagram showing Raman, SECM and ellipsometry as methods of characterising twist angle of twisted bilayer graphene. Top row: optical microscope image of graphene flake, with annotated region of interest that is being characterised. Middle row: single measurement of region of interest (highlighted) using Raman, SECM and ellipsometry. Bottom row: mapping measurement of larger region using Raman, SECM and ellipsometry with approximate time required to extract twist angle information.*

Bilayer Graphene on Cu and Si

When the substrate or dielectric environment of the bilayer graphene is changed the values of ellipsometric angles (e.g. P, A, C and AOI) required to achieve maximum contrast might also be modified, as detailed in previous work.[8] It is therefore equally possible and fast to extract absorption/reflectance spectra on a range of substrates, including on the Cu that the CVD graphene is grown on. SECM becomes particularly powerful when using non-contrast enhancing substrates, where there is often little to no visibility of graphene in conventional optical microscopes (**Figure S3**a,b).[9] We have optimised the contrast of bilayer graphene to demonstrate SECM of bilayer graphene transferred to Si with a native oxide, and as-grown on Cu substrates.

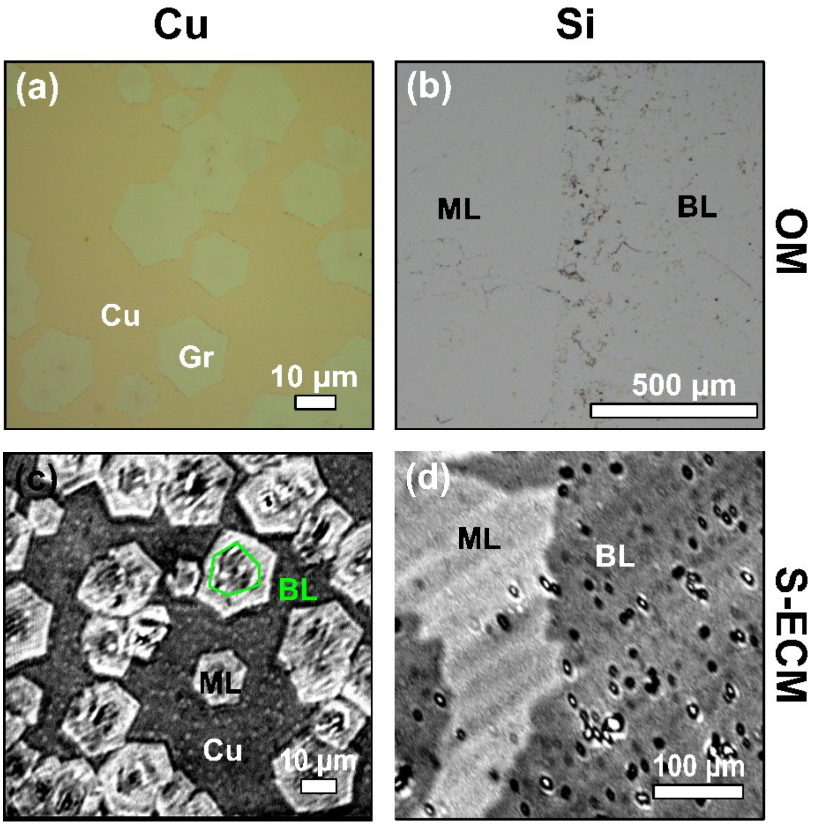

Figure S3*: A demonstration of SECM of as-grown monolayer (ML) and bilayer (BL) graphene on a Cu catalyst as well as on Si(100) polished wafer. We show optical microscope (OM) images (a, b), SECM images (c, d) retrieved as described in the main text, highlighting the enhanced contrast using SECM. The parameters for these measurements were λ=440 nm, P=150°, A=155°, C=45° and AOI = 60° for Si, and λ=400 nm, P=40°, A=80°, C=0° and AOI = 45° for Cu.*

References

(1) Burton, O. J.; Massabuau, F. C. P.; Veigang-Radulescu, V. P.; Brennan, B.; Pollard, A. J.; Hofmann, S. Integrated Wafer Scale Growth of Single Crystal Metal Films and High Quality Graphene. *ACS Nano* **2020**, *14* (10), 13593–13601. https://doi.org/10.1021/acsnano.0c05685.

(2) Geisenhof, F. R.; Winterer, F.; Seiler, A. M.; Lenz, J.; Martin, I.; Weitz, R. T. Interplay between Topological Valley and Quantum Hall Edge Transport. *Nat. Commun.* **2022**, *13* (1), 4187. https://doi.org/10.1038/s41467-022-31680-y.

(3) Medjanik, K.; Fedchenko, O.; Chernov, S.; Kutnyakhov, D.; Ellguth, M.; Oelsner, A.; Schönhense, B.; Peixoto, T. R. F.; Lutz, P.; Min, C.-H.; et al. Direct 3D Mapping of the Fermi

Surface and Fermi Velocity. *Nat. Mater.* **2017**, *16* (6), 615–621. https://doi.org/10.1038/nmat4875.

(4) Keunecke, M.; Reutzel, M.; Schmitt, D.; Osterkorn, A.; Mishra, T. A.; Möller, C.; Bennecke, W.; Jansen, G. S. M.; Steil, D.; Manmana, S. R.; et al. Electromagnetic Dressing of the Electron Energy Spectrum of Au(111) at High Momenta. *Phys. Rev. B* **2020**, *102* (16), 161403. https://doi.org/10.1103/PhysRevB.102.161403.

(5) Keunecke, M.; Möller, C.; Schmitt, D.; Nolte, H.; Jansen, G. S. M.; Reutzel, M.; Gutberlet, M.; Halasi, G.; Steil, D.; Steil, S.; et al. Time-Resolved Momentum Microscopy with a 1 MHz High-Harmonic Extreme Ultraviolet Beamline. *Rev. Sci. Instrum.* **2020**, *91* (6). https://doi.org/10.1063/5.0006531.

(6) Schmitt, D.; Bange, J. P.; Bennecke, W.; AlMutairi, A.; Meneghini, G.; Watanabe, K.; Taniguchi, T.; Steil, D.; Luke, D. R.; Weitz, R. T.; et al. Formation of Moiré Interlayer Excitons in Space and Time. *Nature* **2022**, *608* (7923), 499–503. https://doi.org/10.1038/s41586-022-04977-7.

(7) Ulstrup, S.; Koch, R. J.; Schwarz, D.; McCreary, K. M.; Jonker, B. T.; Singh, S.; Bostwick, A.; Rotenberg, E.; Jozwiak, C.; Katoch, J. Imaging Microscopic Electronic Contrasts at the Interface of Single-Layer WS 2 with Oxide and Boron Nitride Substrates. *Appl. Phys. Lett.* **2019**, *114* (15), 151601. https://doi.org/10.1063/1.5088968.

(8) Braeuninger-Weimer, P.; Funke, S.; Wang, R.; Thiesen, P.; Tasche, D.; Viöl, W.; Hofmann, S. Fast, Noncontact, Wafer-Scale, Atomic Layer Resolved Imaging of Two-Dimensional Materials by Ellipsometric Contrast Micrography. *ACS Nano* **2018**, *12* (8), 8555–8563. https://doi.org/10.1021/acsnano.8b04167.

(9) Blake, P.; Hill, E. W.; Castro Neto, A. H.; Novoselov, K. S.; Jiang, D.; Yang, R.; Booth, T. J.; Geim, A. K. Making Graphene Visible. *Appl. Phys. Lett.* **2007**, *91* (6), 63124. https://doi.org/10.1063/1.2768624.
